# A phase II study of nab-paclitaxel plus carboplatin in combination with thoracic radiation in patients with locally advanced non–small-cell lung cancer

**DOI:** 10.1093/jrr/rrv062

**Published:** 2015-10-05

**Authors:** Takaaki Hasegawa, Yohei Futamura, Akane Horiba, Tsutomu Yoshida, Toshitaka Suzuki, Tatsuo Kato, Daizo Kaito, Yasuhi Ohno, Takayoshi Iida, Shinya Hayashi, Toshiyuki Sawa

**Affiliations:** 1 Department of Respiratory Medicine and Medical Oncology, Gifu Municipal Hospital, 7-1 Kashima-cho, Gifu-shi, Gifu 500-8323, Japan; 2 Respirtory Medicine, National Hospital Organization Nagara Medical Center, 1300-7, Nagara, Gifu-shi, Gifu 502-8558, Japan; 3 Department of Respirology, Gifu University Graduate School of Medicine, 1-1 Yanagido, Gifu-shi, Gifu 501-1194, Japan; 4 Department of Radiology, Gifu Municipal Hospital, 7-1 Kashima-cho, Gifu-shi, Gifu 500-8323, Japan; 5 Department of Radiology, Gifu University Graduate School of Medicine, 1-1 Yanagido, Gifu-shi, Gifu 501-1194, Japan

**Keywords:** non–small-cell lung cancer, nab-paclitaxel, carboplatin, radiotherapy

## Abstract

We investigated the efficacy and safety of albumin-bound paclitaxel (nab-PTX) and carboplatin (CBDCA) with concurrent radiotherapy for unresectable locally advanced non–small-cell lung cancer (NSCLC). Patients with Stage III NSCLC and an Eastern Cooperative Oncology Group performance status of 0 or 1 were eligible. Concurrent chemoradiotherapy consisted of weekly administration of nab-PTX (40 mg/m^2^) plus CBDCA (area under the plasma concentration time curve (AUC) 2) and thoracic radiotherapy (60 Gy/30 fractions) for a total of 6 weeks. After concurrent chemoradiotherapy, patients received an additional two cycles of consolidation phase chemotherapy that consisted of 4-week cycles of nab-PTX (100 mg/m^2^ on Days 1, 8 and 15)/CBDCA (AUC 5 mg/ml/min on Day 1). Response was evaluated in accordance with the Response Evaluation Criteria in Solid Tumors. Progression-free survival and overall survival were estimated using the Kaplan–Meier method. Toxicity was graded using the National Cancer Institute Common Terminology Criteria for Adverse Events. A total of 10 patients were enrolled in this trial between September 2013 and January 2014 from three institutes. The overall response rate was 40.0% and the median progression-free survival was 6.7 months. Treatment-related death occurred in two patients. Grade 2 or worse severe radiation pneumonitis was observed in all three patients that had the volume of lung receiving at least 20 Gy (V20) >30%. The results of this study indicate that no further investigation is warranted into nab-PTX and CBDCA with concurrent thoracic radiation for Stage III NSCLC with V20 > 30% due to severe toxicity.

## INTRODUCTION

Approximately 30% of non–small-cell lung cancer (NSCLC) cases are diagnosed with Stage III disease [1]. A combination of chemotherapy and radiotherapy has been reported to significantly prolong survival compared with radiation therapy alone for unresectable locally advanced NSCLC [2, 3]. Chemoradiotherapy is the standard treatment for locally advanced NSCLC. However, since the median survival time (MST) has been reported as 16.6–26.8 months, treatment development is urgently needed [4, 5].

Albumin-bound paclitaxel (nab-PTX) is a paclitaxel (PTX) formulation in which nanoparticles of PTX are bound to human serum albumin. Because this formulation does not use the solvent that is used for the conventional PTX formulation (polyoxyethylene castor oil and ethanol), this formulation can be administered to alcohol-hypersensitive patients. This is in contrast to the previous formulation that required a longer infusion time and dosing of essential steroids and antihistamines to prevent hypersensitivity.

Combination therapy of carboplatin (CBDCA) and nab-PTX is a useful choice as first-line therapy for patients with advanced NSCLC [6].

Weekly administration of nab-PTX (40 mg/m^2^) plus CBDCA (area under the plasma concentration time curve (AUC) 2) with concurrent radiotherapy (66 Gy) has been reported to be a safe and well-tolerated treatment for locally advanced NSCLC [7].

We conducted a multicenter, phase II study to evaluate the efficacy and safety of this treatment.

## MATERIALS AND METHODS

### Patients

Patients with histologically or cytologically confirmed NSCLC with unresectable Stage IIIA or IIIB disease from three institutes were assessed for eligibility. All patients were required to meet the following criteria: age ≥20; an Eastern Cooperative Oncology Group (ECOG) performance status of 0 or 1; measurable disease; no previous treatment for NSCLC; a leukocyte count of ≥4000/μl; a neutrophil count of ≥2000/μl; a platelet count of ≥100 000/μl; a hemoglobin level of ≥9.5 g/dl; a serum bilirubin level of ≤1.2 mg/dl; serum aspartate aminotransferase and alanine aminotransferase levels of ≤100 IU/ml; a creatinine level of ≤1.2 mg/dl; a blood gas oxygen level of ≥70 Torr in room air; a relative volume of normal lung receiving a dose of ≥20 Gy (V20) ≤35%; and approval of a radiotherapist based on computed tomography (CT) simulation guided by the restrictions of this protocol before registration.

For staging, all patients underwent a CT scan of the thorax and upper abdomen, and either a brain CT scan or brain magnetic resonance imaging. A radioisotopic bone scan or a positron emission tomography (PET) scan was also performed for all patients.

Patients were excluded for any of the following: other active, invasive malignancies in the 5 years leading up to protocol entry; active infections requiring systemic treatment; pulmonary fibrosis; a serious concomitant disease; Grade 2 or higher peripheral neuropathy; positive HBs antigen and/or HCV antibody; history of hypersensitivity for albumin-containing agents; pregnant females; possibly pregnant females; females wishing to become pregnant; nursing mothers; and males currently attempting to produce a pregnancy.

### Ethics, consent and permissions

Written informed consent was required from all patients and the protocol was approved by the institutional ethics committee of each participating institute. This study is registered with the University Hospital Medical Information Network in Japan (UMIN 000011681).

### Treatment schedule

Concurrent chemoradiotherapy was undertaken, with the agents administered at reduced doses weekly for 6 weeks, followed by full-dose chemotherapy during the consolidation phase. The consolidation phase chemotherapy, which was initiated 4 weeks after the concurrent chemoradiotherapy, was administered in two cycles. Thoracic radiotherapy was initiated on Day 1 at a dose of 2.0 Gy daily, five times per week. The total dose of 60 Gy was given in 30 fractions over a 6-week period.

The concurrent phase chemotherapy consisted of nab-PTX (40 mg/m^2^) followed by carboplatin (AUC) 2 mg/ml/min. The consolidation phase chemotherapy consisted of 4-week cycles of nab-PTX (100 mg/m^2^ on Days 1, 8 and 15)/CBDCA (AUC 5 mg/ml/min on Day 1).

### Radiation therapy

All patients were treated with a linear accelerator photon beam of ≥6 MV. The primary tumor and involved nodal disease received 60 Gy in 2-Gy fractions over 6 weeks. A 3D treatment-planning system was used. The initial 40 Gy was delivered to clinical target volume 1 (CTV1), and the final 20 Gy was delivered to a reduced volume defined as clinical target volume 2 (CTV2). CTV1 included the primary tumor, ipsilateral hilum, and mediastinal nodal area from the paratracheal (no. 2) to the subcarinal lymph nodes (no. 7). For primary tumors and involved lymph nodes of ≥1 cm in the shortest diameter, a margin of ≥0.5 cm was added. CTV2 included only the primary tumor and involved lymph nodes, with a margin of 0.5 cm. The spinal cord was excluded from the fields for CTV2 by appropriate methods. An appropriate planning-target-volume margin and leaf margin were added for CTV1 and CTV2. When Grade 4 hematologic toxicity, Grade 3 or 4 esophagitis or dermatitis, pyrexia ≥38.0°C, or Grade ≥2 pneumonitis occurred, the thoracic radiation therapy was interrupted. The V20 was constrained to 35%. The spinal cord dose was constrained to 52 Gy, and the volume of the spinal cord to be irradiated by >48 Gy was set to ≤1 cm^3^. The maximum dose per fraction of spinal cord should not exceed 2.3 Gy.

### Evaluation of response and toxicity

All eligible patients received the following evaluations: chest X-rays, complete blood cells, and blood chemistry studies were repeated once a week during the treatment period. Thoracic CT was performed after the concurrent chemoradiotherapy. After the treatment, thoracic CT was performed every 6 months, and other imaging examinations were performed when recurrence was suspected. The response was evaluated in accordance with the Response Evaluation Criteria in Solid Tumors, version 1.1. Progression-free survival (PFS) was defined as the time from registration until objective tumor progression or death. Overall survival (OS) was defined as the time from registration to death from any cause. PFS and OS were estimated by the Kaplan–Meier method. Toxicity for all patients was assessed and graded by using the National Cancer Institute Common Terminology Criteria for Adverse Events, version 4.0.

### Statistical analysis

The primary endpoint of this study was the objective tumor response rate. For the first stage, Simon's minimax two-stage phase II design was used to allow early termination if preliminary results indicated minimal efficacy. We choose a 75% response rate as the experimental target level and a 50% response rate as the minimal target level, with a one-sided α-error of 0.025 and a β-error of 0.1, resulting in a target of 13 patients. If responses of ≥7 patients were observed among the 13 total assessable patients, the treatment was considered worthy of further consideration. For the second stage, an additional 17 patients were enrolled. Therefore, a total of 32 patients was the planned accrual size because of the possibility of including ineligible patients. Statistical analyses were performed using SPSS version 21.0 (IBM Corp., Armonk, NY).

## RESULTS

### Patient characteristics

A total of 10 patients were enrolled in the trial between September 2013 and January 2014 from three institutes. Patient characteristics are summarized in Table 1. The median age was 73 years. The ECOG performance status was 0 for 30% of patients and 1 for 70% of patients. Of these patients, 50% had squamous cell carcinoma and 50% had adenocarcinoma. The median V20 was 22%. All but one of these patients completed the concurrent phase chemotherapy. Because of Grade 5 lung infection, one patient was unable to complete the scheduled concurrent phase chemotherapy.

**Table 1.** Patient characteristics

Patient characteristics	*n*
No. of eligible patients	10
Age, y	
Median (range)	73 (57–80)
Gender	
Male	9
Female	1
ECOG PS	
0	3
1	7
Smoking history	
Absent	1
Present	9
Histology	
Squamous cell carcinoma	5
Adenocarcinoma	5
Stage	
IIIA	5
IIIB	5
Location of primary site	
Upper lobe	5
Middle lobe	0
Lower lobe	5
V20, %	
Median (range)	22 (17–35)

ECOG = Eastern Cooperative Oncology Group, PS = performance status, V20 = %, percentage of lung receiving >20 Gy.

### Response to treatment

We observed that 40% of all patients had a partial response (PR) and 50% had stable disease. No patient had a complete response or progressive disease. The overall response rate was 40.0% (95% Confidence Interval (CI): 12.1–73.7).

### Toxicity of the treatment

Major Grade ≥3 severe toxicities for this treatment are summarized in Table 2. A total of seven patients were unable to complete the consolidation phase chemotherapy because of toxicities (radiation pneumonitis, lung infection, or heart failure), poor PS, or patient preference. The most common Grade 3/4 hematological toxicity was leukopenia (eight patients, 80%). Other Grade 3/4 hematological toxicities were neutropenia (five patients, 50%) and anemia (one patient, 10%). Other Grade ≥3 severe toxicities were anorexia (three patients, 30%), nausea (two patients, 20%), diarrhea (one patient, 10%), radiation pneumonitis (two patients, 20%), heart failure (two patients, 20%) and lung infection (one patient, 10%). The grading of the lung infection was 5; it was developed by occlusion of the tumor. Obstructive pneumonitis was a complication of a primary lesion, we had determined that it was not treatment-related death.

**Table 2.** Hematological and non-hematological major adverse events

CTCAE version 4	G1/2	G3/4	G5	All grades (%)	≥G3 (%)
Leukopenia	0/2	6/2	0	100	80
Neutropenia	0/4	3/2	0	90	50
Thrombocytopenia	7/3	0/0	0	100	0
Anemia	3/6	1/0	0	100	10
Blood bilirubin increased	1/0	0/0	0	10	0
AST abnormalities	2/0	0/0	0	20	0
ALT abnormalities	2/0	0/0	0	20	0
Creatinine increased	1/0	0/0	0	10	0
Anorexia	4/1	3/0	0	80	30
Nausea	4/2	2/0	0	80	20
Vomiting	4/0	0/0	0	40	0
Diarrhea	1/0	1/0	0	20	10
Fatigue	5/3	0/0	0	80	0
Alopecia	2/3	0/0	0	50	0
Neuropathy	2/0	0/0	0	20	0
Myalgia	4/0	0/0	0	40	0
Arthritis	2/1	0/0	0	30	0
Radiation pneumonitis	2/3	1/0	1	70	20
Weight loss	1/4	0/0	0	50	0
Heart failure	0/0	1/0	1	20	20
Esophagitis	2/1	0/0	0	30	0
Lung infection	0/0	0/0	1	10	10
Febrile neutropenia	0/0	0/0	0	0	0

ALT = alanine transaminase, AST = asparate transaminase, CTCAE = Common Terminology Criteria for Adverse Events.

Table 3 summarizes the grading of radiation pneumonitis and V20. Grade ≥2 severe radiation pneumonitis was observed in all three patients that had V20 > 30%, and one of them died of Grade 5 radiation pneumonitis.

**Table 3.** Grade 3 or worse severe pneumonitis according to V20

V20	*n* (%)
<20% (*n* = 4)	1 (25)
20–29.9% (*n* = 3)	0 (0)
30.0–35.0% (*n* = 3)	1 (33.3)

### Survival

For the current analysis, the overall median follow-up time was 8.2 months (range, 1.3–10.9). The median PFS was 6.7 months (95% CI: 0.6–12.9). Treatment-related death occurred in two patients. One patient died of radiation pneumonitis. A 72-year old man with a smoking history, a V20 of 33% and a performance status of 1 presented with a cough and fever on Day 104 from the initiation of chemoradiotherapy. A CT scan showed bilateral ground-glass opacity; no bacterial infection was observed in bronchoscopy. He received methylprednisolone pulse therapy and cyclophosphamide pulse therapy, but he died of respiratory failure as a result of radiation pneumonitis on Day 124. Another patient died of heart failure. A 58-year-old woman with a smoking history, a V20 of 18% and a performance status of 1 presented with cough and dyspnea on Day 175 from the initiation of chemoradiotherapy. A CT scan showed bilateral consolidation, pleural effusion and pericardial effusion, without coronary artery calcification. An echocardiogram showed an ejection fraction of 23%. Therapy was initiated with prednisolone therapy at 0.5 mg/kg per day, and the infiltrative shadow on radiography gradually improved. However, she died of heart failure on Day 260 with Grade 3 radiation pneumonitis. Two patients had a non-related death; one patient died of a lung infection and the other patient died with disease progression. This study was discontinued due to these adverse events.

## DISCUSSION

All but one of these patients completed nab-PTX and CBDCA chemotherapy with concurrent radiotherapy. During the concurrent phase chemotherapy, patients had mild toxicities and no febrile neutropenia was observed. However, the incidence of radiation pneumonitis and other adverse events that occurred after the concurrent phase were not acceptable. Furthermore, the response rate was lower than expected, although all but one of these patients displayed partial response or stable disease.

For unresectable locally advanced NSCLC, it is recommended to perform chemoradiotherapy that includes platinum. For chemotherapy in combination with radiation, the combination use of PTX and CBDCA, or of docetaxel and cisplatin, is the standard treatment, and this therapy has been reported on in two Phase III trials in Japan [4, 8]. However, for either of these combination therapies, complete non-inferiority or superiority to chemotherapy consisting of vindesine, cisplatin and mitomycin has not been shown, and therefore further study is needed to determine the optimal chemotherapy. A study by Desai *et al.* using nude mice suggested that nab-PTX increased antitumor activity and intratumoral PTX concentrations more than cremophor-based PTX when an equal dose was delivered [9]. There is a previous report by Sparreboom *et al.* indicating that the amount of nab-PTX distributed to the lungs of rats was higher than that of PTX 24 h after administration, but that its distribution to the lungs was lower than that of PTX after 120 h. This distribution of nab-PTX may possibly reduce pulmonary toxicity [10].

Based on these data, nab-PTX is a promising drug. This is the first Phase II study to investigate the combined use of nab-PTX and CBDCA in a curative setting for Stage III NSCLC.

In the Phase I trial, no pneumonitis was observed [7]. That study reported that chemotherapy consisting of nab-PTX (40 mg/m^2^) followed by CBDCA (AUC 2 mg/ml/min) with thoracic radiotherapy was safe and well tolerated, with adverse events that were expected for concurrent chemoradiotherapy. Furthermore, no pneumonitis was observed in the Phase III trial of advanced-stage NSCLC [6]. However, the incidence of radiation pneumonitis was significantly higher in our study than in those studies. In a past meta-analysis, elderly age, high V20 value and CBDCA–PTX chemotherapy in chemoradiation therapy were reported as predictors of radiation pneumonitis [11]. There is a previous report by Dang *et al.* indicating that response to chemoradiotherapy treatment with concurrent docetaxel/cisplatin or vinorelbine/cisplatin was found to be a predictor for Grade ≥3 radiation pneumonitis [12]. Therefore, the fact that the chemotherapy that we used in our study was a taxane regime was a risk factor for the occurrence of radiation pneumonitis. The rate of severe radiation pneumonitis that developed in cases with high V20 or PR was consistent with these reports. Omitting elective nodal irradiation during thoracic irradiation may reduce the incidence of radiation pneumonitis.

On the other hand, one patient died of Grade 5 heart failure. She had left upper-lobe primary squamous cell carcinoma and multiple metastases of mediastinal and ipsilateral hilum lymph nodes. The chemotherapy that we used in our study was a taxane regime; that and the irradiation dose to the heart were considered to be risk factors for the occurrence of cardiotoxicity [13, 14]. This patient's treatment included a taxane regime and a higher dose of mediastinal irradiation, which might have influenced the development of heart failure.

There have been very few reports of combination therapy of nab-PTX and radiation therapy. De Petris *et al.* reported that gross tumor volume (GTV) plays a prognostic role in NSCLC [15]. In the present study, a large number of cases had a high V20 value. Therefore, if some cases of large GTV had been included in this study, the results might have indicated poor prognosis. The present study was an analysis of 10 cases only. Since the consolidation phase of chemotherapy was carried out for very few patients, the response rate observed is used for reference only. It is possible that this treatment might yet become an effective therapeutic strategy. The most important point of this study was the introduction of new knowledge regarding such treatment.

Many of the patients completed nab-PTX and CBDCA with concurrent radiotherapy. In order to implement this treatment regimen, it is necessary to keep V20 < 30% using methods such as omitting elective nodal irradiation during thoracic irradiation. When carrying out this study treatment in the future, it may be limited by V20 or the trial protocol; avoidance of cases at risk of radiation pneumonitis is desired. It may be useful to examine the degree of expression of secreted protein acidic and rich in cysteine (SPARC) in the cancer cells of patients who develop pneumonitis, because SPARC expression has been shown to correlate with poor prognosis for NSCLC patients [16].

## FUNDING

No financial support was received for this study. Funding to pay the Open Access publication charges for this article was provided by Gifu Municipal Hospital.
